# A Structure-Based Mechanism for the Denaturing Action of Urea, Guanidinium Ion and Thiocyanate Ion

**DOI:** 10.3390/biology11121764

**Published:** 2022-12-05

**Authors:** Antonella Paladino, Nicole Balasco, Luigi Vitagliano, Giuseppe Graziano

**Affiliations:** 1 Institute of Biostructures and Bioimaging, CNR, Via Pietro Castellino 111, 80131 Naples, Italy; 2 Institute of Molecular Biology and Pathology, CNR c/o Department Chemistry, Sapienza University of Rome, P. le A. Moro 5, 00185 Rome, Italy; 3 Department of Science and Technology, University of Sannio, Via Francesco de Sanctis snc, 82100 Benevento, Italy

**Keywords:** urea, protein stability, urea-protein interactions, chemical denaturants

## Abstract

**Simple Summary:**

The detailed characterization of urea binding sites in protein structures shows that urea can establish multiple types of interactions, in line with recent findings reported for guanidinium and thiocyanate, thus confirming that promiscuity is a general property of protein denaturants. Our analyses support a denaturing model based on protein-denaturant direct interactions to practically equal and independent sites. We also underscore insightful features that can inform on the milder denaturing power displayed by urea.

**Abstract:**

An exhaustive analysis of all the protein structures deposited in the Protein Data Bank, here performed, has allowed the identification of hundredths of protein-bound urea molecules and the structural characterization of such binding sites. It emerged that, even though urea molecules are largely involved in hydrogen bonds with both backbone and side chains, they are also able to make van der Waals contacts with nonpolar moieties. As similar findings have also been previously reported for guanidinium and thiocyanate, this observation suggests that promiscuity is a general property of protein denaturants. Present data provide strong support for a mechanism based on the protein-denaturant direct interactions with a denaturant binding model to equal and independent sites. In this general framework, our investigations also highlight some interesting insights into the different denaturing power of urea compared to guanidinium/thiocyanate.

## 1. Introduction

The elucidation of the physico-chemical factors that drive protein folding is a puzzling and largely unsolved issue that has attracted the attention of the scientific community since the determination of the first three-dimensional structure of a globular protein, more than sixty years ago [1]. The difficulties encountered in this field are related to the observation that the folded structure of a protein is marginally more stable in comparison to the ensemble of its unfolded states [2,3]. Although the development of machine learning algorithms—that in many cases provide reliable three-dimensional models of proteins starting from their sequences, represents a revolution in structural biology with unforeseeable consequences—many fundamental aspects of protein folding remain obscure [4]. These include the definition of structure-stability relationships and the interpretation, at the molecular level, of the plethora of experimental data on the impact that chemical denaturants have on protein folding.

It is known for a long time that the conformational stability of globular proteins can be strongly affected by the addition to water of certain chemical species that prove to have a denaturing action toward the folded state [5]. Among them, urea (Figure S1) and guanidinium chloride are the most used in labs all over the world to determine protein stability in terms of folding/unfolding Gibbs free energy change. Notwithstanding the incredibly huge number of experimental studies reporting denaturant-induced unfolding data, the mechanism of action of chemical denaturants has not yet been clarified. One of the reasons is the weakness of the denaturing effect of these chemical species: the concentration of urea or GdmCl necessary to unfold a stable globular protein at room temperature is around 2–6 M [6], to be contrasted with a protein concentration of about 10^−4^ M. Similarly, the denaturation temperature of RNase A at pH 7.0 is 63.4 °C in aqueous buffer solution, 59.7 °C in 1 M urea, 53.9 °C in 1 M GdmCl, and 47.9 °C in 0.5 M GdmSCN [7]. This weakness can be understood by considering that chemical denaturants have to replace a fraction of water molecules covering the protein surface, and by recognizing that the density of this water monolayer is higher than that of bulk water [8,9]. In fact, Schellman used the expression “solvent denaturation” [10] to name the denaturation induced by urea and GdmCl. Therefore, there is still controversy between a direct mechanism, implying direct attractive interactions of chemical denaturants with protein surfaces, and an indirect mechanism, implying a modification of water properties that cause a decrease in the magnitude of the hydrophobic effect, the main determinant of the folded state stability [11,12] (it is not our aim to perform a general survey of the matter, and so only a small selection of published articles has been cited) [7,13,14,15,16,17,18,19,20,21].

Denaturant-induced unfolding curves, at a constant temperature, are usually analyzed by means of the linear extrapolation model, LEM, or the denaturant binding model, DBM [22,23,24,25,26]. The first one does not assume any mechanism of action of the denaturing agent, but solely that the folding-unfolding Gibbs free energy change depends linearly on the denaturant concentration. The second one assumes that the denaturing agent binds on the protein surface to equal and independent sites, shifting the equilibrium toward the unfolded state that possesses a greater number of binding sites because of its larger water-accessible surface area, WASA [27]. Both models seem to work well in fitting experimental data, so they have not been useful to clarify the mechanism of action of chemical denaturing agents.

In the last few years, we have approached the matter in a different manner. We have performed a search over all the protein structures deposited in the Protein Data Bank, PDB, to try to observe the presence of bound guanidinium ions and thiocyanate ions. Somewhat unexpectedly, it has been possible to detect 127 different binding sites for guanidinium ion (Gdm^+^) [28], and 712 different binding sites for thiocyanate ion (SCN^−^) [29], and to perform an in-depth analysis of the occurring contacts and interactions. In the present study we extend the analysis: (1) to observe the presence of bound urea molecules in all the protein structures deposited in the PDB; (2) to make a comparison between the binding sites of Gdm^+^, SCN^−^ and urea; (3) to provide structural evidence and thermodynamic explanation of the validity of a mechanism accounting for the binding of these species on protein surfaces; and (4) to highlight, in this general framework, the analogies and differences in the physico-chemical behavior of these three commonly used denaturants.

## 2. Materials and Methods

### 2.1. Analysis of the Protein Data Bank and Selection of Urea Binding Sites

The identification of independent binding sites of urea in PDB protein structures and the analysis of the interactions that stabilize these adducts were here performed by adapting the protocol previously developed for Gdm^+^ and SCN^−^ denaturing agents [28,29]. Specifically, protein structures containing urea were identified by interrogating the entire PDB release of May 2022, using as a query the three-letter code URE, which denotes this ligand in this data bank.

The approach led to the identification of 78 entries in which urea is present as a standalone ligand. Of these, one entry was excluded as it contains urea interacting exclusively with RNA, in a protein-RNA complex (PDB entry: 4qg3).

As these structures could contain multiple copies of the ligand, they were initially classified in terms of the number of urea molecules present in the asymmetric unit. Moreover, as the PDB frequently contains multiple entries of the same protein, generally determined in different experimental conditions, to avoid redundancy, for each protein we selected the one containing the highest number of urea molecules in the asymmetric unit (Table S1). In the case of multiple entries for the same protein containing the same number of urea molecules, the structure determined at the highest resolution was considered.

As reported in Table S1, the urea ligand has been detected in the structures of 47 different proteins. In the case of Arginase, two entries (PDB entry: 6nfp and 1hqg) have been considered in the analysis since these two protein variants were isolated from different species (*Bacillus subtilis* and *Rattus norvegicus*, respectively), and are characterized by a limited level of similarity (42.2% sequence identity). Application of this protocol led to the selection of a total of 320 bound urea ligands, distributed in these 47 proteins (see urea in A.U. of Table S1).

As shown in Table S1, in fourteen of these 47 selected entries the asymmetric unit contains multiple copies of the biological assembly. When in these copies the urea ligand(s) was bound to identical protein patches, to avoid redundancy, we considered only a single representative example. A similar approach was used for multimeric proteins. Indeed, if the urea was bound to a similar/identical environment in the different chains of the oligomer, we selected only one representative example. After this additional selection, we ended up with a total of 289 independent bound urea ligands (see independent urea in Table S1). It is important to note that the urea molarity used in these crystal structures spans a large concentration range, up to 9 M (Table S1 and Figure S2).

### 2.2. Analysis of Urea-Protein Interactions

The interactions established by urea molecules with the protein residues, cofactors, ions, and other entities present in the PDB entries were computed using the LigPlot+ software [30], together with manual inspections of the individual PDB structures. Therefore, we selected interactors and interactions using the criteria adopted by LigPlot+: (i) a distance within 3.35 Å for H-bonding; and (ii) a distance within 3.90 Å for the interactions with the aliphatic/aromatic groups. As LigPlot+ does not consider the interactions of the ligand with symmetry-related mates in the crystalline state, we individually inspected all selected entries using COOT [31] and classified these interactions following the LigPlot+ criteria. Therefore, for each urea molecule the interactors identified manually or by LigPlot+ were collectively considered. As reported in Table 1, the interactions were classified into H-bonds (with main chain or side chain atoms), van der Waals-type contacts with aliphatic/aromatic groups, and inter-ligand (urea-urea) contacts. Interactions with cofactors/ions/other ligands were also analyzed (Table 1).

Figures were generated using VMD [32] and plots were produced by XMGRACE [33].

## 3. Results

### 3.1. Overview of Urea Binding Sites

**Figure 1:**
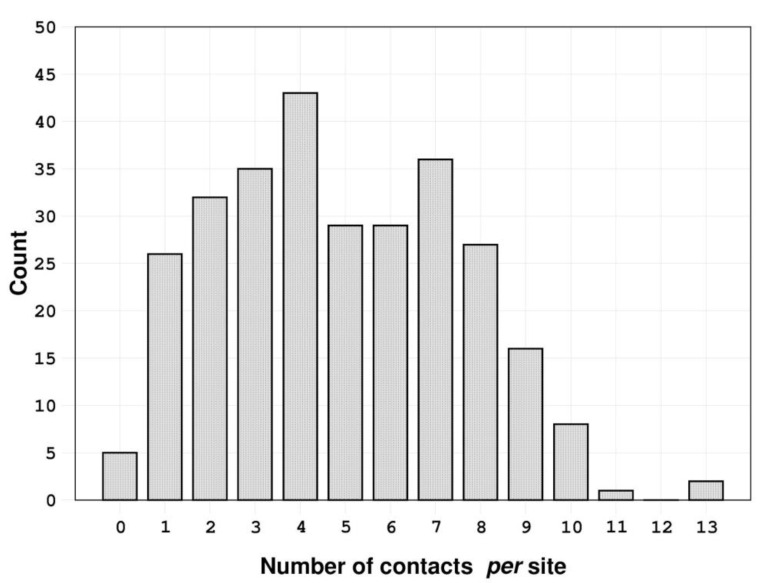
Distribution of the number of contacts *per* site established by the 289 urea molecules analyzed.

**Figure 2:**
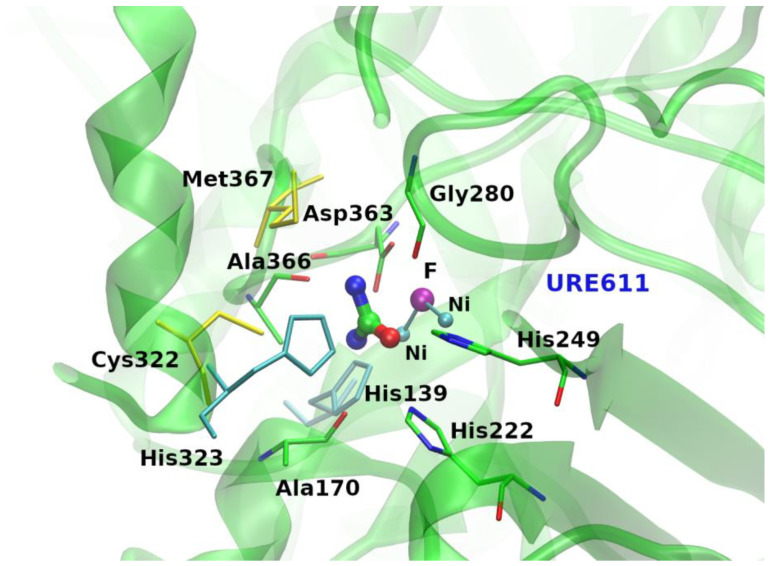
Binding site of the *Sporosarcina pasteurii* urease protein (PDB ID: 6qdy [35]) where urea makes the largest number of contacts. Hydrogen bonds with main and side chains of both aliphatic and hydrophobic amino acids, nonpolar interactions with protein atoms as well as with ions stabilize urea (URE611) in the present binding site. Yellow and cyan sticks represent aliphatic and aromatic van der Waals-type contacts, respectively.

The analysis of the interactions established by urea molecules in the selected PDB entries (using the protocol described in the Methods section) shows that most of them make intricate and varied networks of contacts with their partners (Figure 1 and Figure S3 and Table S2). Indeed, the identified bound urea molecules can make as many as thirteen atomic contacts (see the Methods section for the definition). The latter very high number of contacts made by urea was detected in the structures of the chemoreceptor TIpB from *Helicobacter pylori* [34] (PDB entry: 3ub6), and of the urease from *Sporosarcina pasteurii* [35] (PDB entry: 6qdy), displayed in Figure 2. In 94.5% of the cases, urea establishes a number of contacts between 1 and 9. Urea is stabilized by a single contact in 26 structures; this single contact may be an aliphatic interaction (15 cases), an H-bond with a water molecule (10 cases), or an H-bond with a residue side chain (one case). On average, protein-urea interactions are mediated by five contacts. Although remarkable, this number is smaller than that previously observed for either Gdm^+^ or SCN^−^ (average value = 6).

A complete survey of the interactions that urea molecules form with their partners in the selected protein structures is reported in Table 1, where, for comparative purposes, also listed are the values obtained from similar investigations carried out on both Gdm^+^ and SCN^−^ ions [28,29]. In addition to the contacts with the protein chain and the solvent molecules, which will be described in detail in the following paragraphs, urea molecules occasionally interact with ions (three chlorides), metals (three Mn, two Ni, one Cu, and one Zn), cofactors and other ligands present in the PDB entries. Moreover, a significant number of urea molecules self-associate by interacting with other urea molecules. The percentage of urea molecules making self-contacts (12.1%) is larger than that observed for Gdm^+^ ions (5.5%). This is not surprising considering the ionic nature of the latter compound and the consequent electrostatic repulsion associated with the self-interaction.

### 3.2. Insights into Urea-Protein Interactions

**Figure 3:**
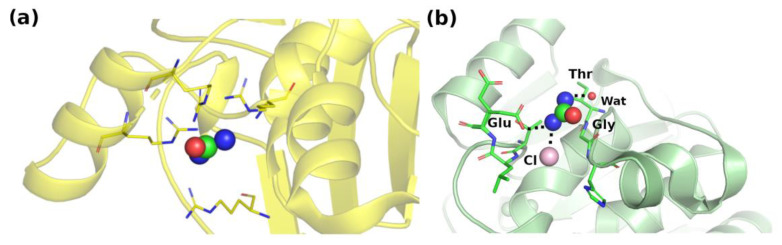
Interaction network where urea makes (**a**) multiple types of contacts with arginines (PDB ID: 5ulp [36]), and (**b**) multiple main-chain and side-chain H-bonds (PDB ID: 5i4y [37]). For clarity, interacting water molecules are omitted in (**a**).

Inspection of Table 1 indicates that urea frequently interacts with its partners by making H-bonds. This is not surprising as this molecule is endowed with groups that can act as hydrogen acceptors or donors in this type of interaction. This dual possibility to form H-bonds is particularly evident in its interaction with the protein chain. Indeed, more than two-thirds of urea molecules form H-bonds with the protein partner, a percentage that is larger than that observed for Gdm^+^ (69% versus 61%), and much larger than that observed for SCN^−^ (69% versus 37%). The H-bonds formed by urea with proteins are equally distributed between the main chain and side chain groups (Figure 3).

**Figure 4:**
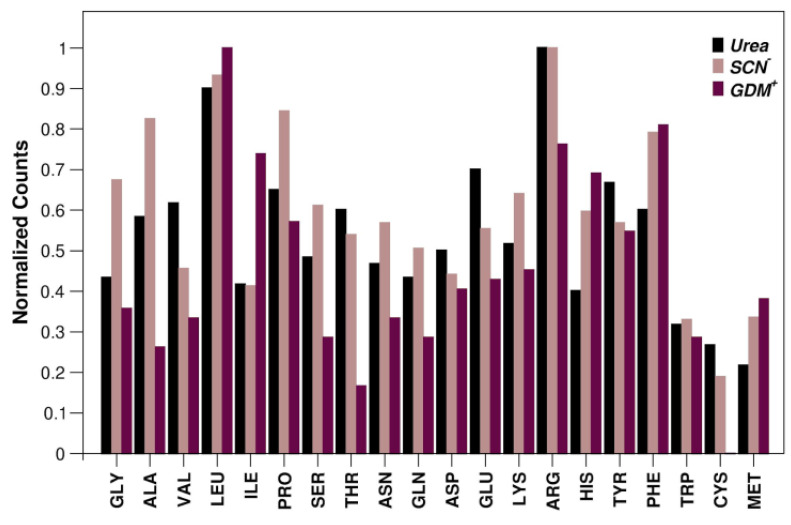
Representativeness of residue type involved in van der Waals-type interactions *per* urea, SCN^−^ and Gdm^+^ sites. Normalized values are given as the ratio of the observed value to the maximum value of the determined distribution. Data corresponding to SCN^−^ are retrieved from reference [29]; distribution referred to Gdm^+^ is generated using database of reference [28]. See also Figure S4.

As found for the other two denaturants, in addition to the expected H-bonding interactions, urea molecules detected in the crystalline structures of proteins frequently make contacts with nonpolar residues. This agrees with the results of an x-ray diffraction study on lysozyme-urea complexes, prepared by soaking protein crystals in a 9 M urea solution for different periods of time [37]. Of particular relevance is the finding that van der Waals contacts, preferentially with aliphatic groups, are observed in the vast majority of the binding sites (Figure 4). The ability of urea to make contacts with both polar and nonpolar groups renders this species able to interact with all protein residues, almost independently of their physico-chemical properties. Indeed, the distribution of interacting residues somehow reflects the amino acid composition in proteomes [38]. Present data are in line with the extensive molecular dynamics study performed by Horinek and Netz, using different force fields for both urea and water [39]. It emerged that urea interacts favorably, from the energetic point of view, with both the backbone and the side chains, regardless of their polar or nonpolar character [39,40].

The residue that makes the highest number of contacts with urea is arginine, as shown in Figure 4. This is due both to its high abundance in protein sequences and to the presence in its side chain of both nonpolar and charged moieties. Interestingly, we detected multiple interactions of urea with the guanidinium group of Arg. Although we could identify several cases where a single urea molecule is surrounded by numerous guanidinium groups from Arg residues or by other urea molecules (Figure 3), there is no clear evidence for a preferential geometric orientation with respect to the planarity of the guanidinium moiety or urea molecules.

Inspection of Table 1 also highlights the reduced tendency of urea to form nonpolar van der Waals interactions in comparison to the behavior of both thiocyanate and guanidinium ions. This is particularly evident by comparing the ability of these denaturants to interact with the π-system of aromatic side chains (Figure S4). A large polarizability is surely a fundamental feature to interact strongly with aromatic rings. The soft thiocyanate ion has a large polarizability, originating in its delocalized charge distribution [41]. Urea molecules, in contrast, are not so polarizable, but possess a very large dipole moment, around 5 Debye, according to quantum chemical calculations in water, and around 10 Debye, according to the analysis of dielectric relaxation spectroscopy measurements in aqueous solution [42]. In this scenario, it is not surprising that purely nonpolar binding sites represent a minimal percentage of the total sites for urea, 8.6%, to be contrasted with 15% for SCN^−^ ions.

### 3.3. Urea Solvation

**Figure 5:**
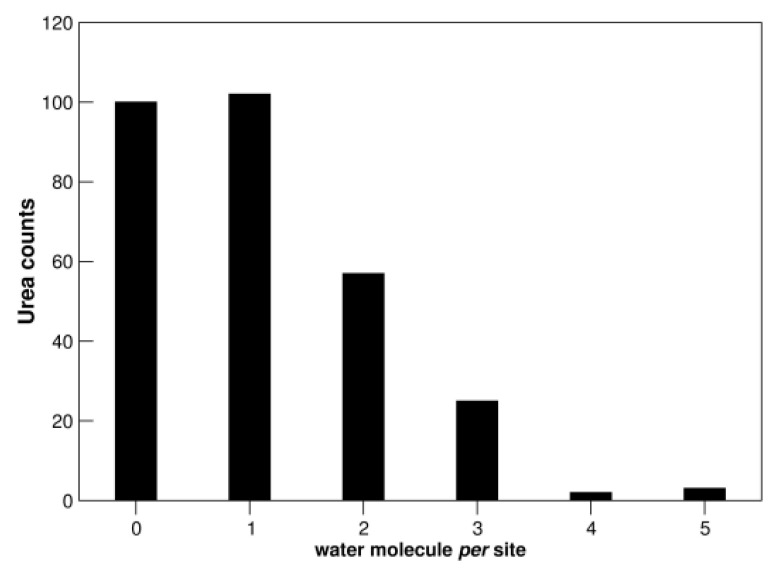
Distribution of water molecules *per* urea binding site. See also Figure S5.

The ability of urea to act as both hydrogen donor and hydrogen acceptor in H-bond formation is largely exploited in its interaction with water molecules (Table 1 and Figure 5). In fact, more than 65% of analyzed urea binding sites are characterized by the presence of at least one water molecule; note, in contrast, that about 50% of the analyzed thiocyanate binding sites do not contain water (Figure S5). Urea molecules use almost indistinctly oxygen (~38%) and nitrogen atoms (~62%) to mediate water interactions. Previous analyses performed on thiocyanate and guanidinium ions highlighted a poorer hydration profile for both ligands (Figure S5), particularly for SCN^−^ [28,29]. These data are in line with firmly established neutron scattering measurements on aqueous solutions of urea [43], Gdm^+^, and SCN^−^ ions [44,45]. These two ions prove to be “poorly” hydrated in water, due to the marked delocalization of the single charge over the entire structure. In contrast, urea proves to be very well hydrated, forming, on average, six H-bonds with surrounding water molecules [43], and can be considered to be a water dimer [43,46]. It is interesting that a urea molecule is usually assumed to replace two water molecules on protein surfaces [20,39], and this assumption is supported by structural data [37]. The strength of urea-water interactions can be one of the reasons rendering urea a poorer denaturant in comparison to Gdm^+^ and SCN^−^ ions.

## 4. Discussion

The environment that a polypeptide chain experiences in the crystalline state may significantly differ from that of aqueous solutions. However, crystals of globular proteins contain a lot of water, and urea can diffuse in their interior to reach the protein surface [37]. This means that a survey of all the available crystal structures with bound urea or Gdm^+^ or SCN^−^ can provide more than reliable hints of what happens in an aqueous solution. Moreover, it is important to recognize that: (1) the addition of either urea, GdmCl, or GdmSCN to water leads to a density increase [7] (i.e., a change in an important bulk water property) that, in turn, causes an increase in the magnitude of the solvent-excluded volume effect (i.e., the magnitude of the reversible work of cavity creation), and the latter stabilizes the folded state [7,18,19,20]; (2) modifications in bulk water structure (i.e., reorganization of water-water H-bonds) cannot be the driving force of anything because an almost complete enthalpy-entropy compensation is operative [47]. These two remarks imply that an indirect mechanism for the denaturing action of urea, Gdm^+^, and SCN^−^ has no physico-chemical grounds.

The analysis of all the protein structures deposited in the PDB indicates unequivocally that Gdm^+^, SCN^−^, and urea have the ability to bind the surface of folded proteins. Since the difference between the surface exposed to water and that buried in the interior is solely related to the charged fraction [48], and no effective preference emerges in the analysis of binding sites, it should be safe to conclude that a greater number of binding sites is available on the surface of unfolded proteins. This means that the denaturing action of such chemical species should mainly be caused by their ability to bind protein surfaces, replacing water molecules. The reliability of this mechanism, inferred from structural data, is supported by the results of several molecular dynamics studies on globular proteins and model systems in aqueous solutions [15,16,17,39,40]. Even though such a scenario is absolutely not new [13,14,15,17,18,19,23,24], a historical tale of the thermodynamic approaches used to analyze experimental data may be important to gain the right perspective.

In an important article published in 1992, Makhatadze and Privalov [49], by performing ITC and DSC measurements on both the folded and unfolded state of three proteins (i.e., RNase A, lysozyme and cytochrome c), characterized the interaction thermodynamics of proteins with urea and GdmCl. The results can be summarized by the following statements: (1) the interaction can accurately be described as the binding of urea molecules or Gdm^+^ ions to equal and independent sites; the number of the latter increases markedly on passing from the folded to the unfolded state; (2) the binding of both urea and Gdm^+^ is exothermic with an average binding enthalpy change *per* site Δh_b_ = (−9 ± 2) kJ mol^−1^ for urea, and (−11 ± 2) kJ mol^−1^ for Gdm^+^; (3) the average binding constant *per* site K_b_ =0.06 M^−1^ for urea, and 0.6 M^−1^ for Gdm^+^ at 25 °C; these small numbers may appear strange, but are in line with the large concentrations of urea and GdmCl usually necessary to unfold globular proteins, and are caused by the need to replace water molecules contacting the protein surface; (4) the corresponding average binding Gibbs free energy *per* site, at 25 °C, Δg_b_ = 6.9 ± 1.0 kJ mol^−1^ for urea, and 1.3 ± 1.0 kJ mol^−1^ for Gdm^+^; the average binding entropy change *per* site, at 25 °C, Δs_b_ = (−53 ± 8) J K^−1^ mol^−1^ for urea, and (−41 ± 8) J K^−1^ mol^−1^ for Gdm^+^; (5) the binding of urea or Gdm^+^ to a single site is not thermodynamically favored because the negative enthalpy change is overwhelmed by a negative and larger entropy change; (6) the overall binding process proves to be thermodynamically favored and destabilizes the folded state because there is a large and positive entropy contribution originating in the configurational disorder associated with the occurrence of occupied and unoccupied binding sites, whose number significantly increases on passing from the folded to the unfolded state. In fact, the basic statistical thermodynamic equations of this model are the following [10,49]:

<ΔGb> = −Δn·RT·ln(1 + Kb[L])(1)

<ΔHb> = Δn·Δhb·{Kb[L]/(1 + Kb[L])}(2)

<ΔSb> = Δn·R·ln(1 + Kb[L]) + (Δn·Δhb/T)·{Kb[L]/(1 + Kb[L])}(3)

where the angled brackets indicate that the contribution of all the binding sites is accounted for, and Δn is the difference in the number of binding sites between the unfolded and the folded state, so that Δn > 0; and, more correctly, the activity should be used instead of molar concentration [22]. It is noteworthy that the binding enthalpy change is totally compensated by one of the two contributions constituting the binding entropy change; this enthalpy-entropy compensation implies that the <ΔG_b_> quantity is purely entropic (i.e., this is a specific feature of the binding to equal and independent sites). Clearly, the association is favored on increasing both the K_b_ value and the ligand concentration [L]. In other words, the equilibrium is shifted toward the unfolded state by increasing the denaturant concentration, even though the binding constant *per* site is smaller than one. For instance, at 25 °C, using the K_b_ value of urea determined by Makhatadze and Privalov [49], and fixing Δn = 20, Equation (1) leads to <ΔG_b_> = −2.9 kJ mol^−1^ at [urea] = 1 M, −5.6 kJ mol^−1^ at [urea] = 2 M, and −8.2 kJ mol^−1^ at [urea] = 3 M. These numbers are reliable in comparison to the conformational stability of globular proteins around room temperature [2,3]. Denaturation would be more favored if the average binding constant *per* site was greater than one, indicative of a preference of the denaturant for a site on the protein surface with respect to the bulk water.

Schellman performed a careful and more complex thermodynamic analysis of experimental data, trying to account explicitly for water replacement associated with denaturant binding to protein surface [50,51]; nevertheless, he concluded that the sites can be described as equal and independent, and the average value of K_b_ *per* site is slightly greater than one for both urea and Gdm^+^. On the other hand, Cremer and colleagues, from the analysis of data related to the shift in the lower critical solution temperature of both Poly(N-isopropylacrylamide), and elastin-like-polypeptides [52,53], obtained that K_b_ *per* site amounts to about 5 M^−1^ for the SCN^−^ ion at room temperature. The latter value, being significantly larger than those determined for urea molecules and Gdm^+^ ions, confirms the well-known stronger denaturant activity of the thiocyanate ion. Record and colleagues [54,55], by analyzing experimental data with their solute partitioning model (accounting for both preferential interaction and water replacement), determined that, at room temperature, the binding constant *per* site amounts to 1.1 M^−1^ for urea, 1.6 M^−1^ for Gdm^+^, and 2.4 M^−1^ for SCN^−^. It can be concluded that different thermodynamic models arrive at the same scenario with similar values.

Present data are in line with the finding that a simple binding model to equal and independent sites, accounting also for water replacement, works well in describing the interaction of urea molecules, Gdm^+^ ions, and SCN^−^ ions with protein surfaces and rationalizing their denaturing effect. These denaturants possess binding sites on protein surfaces that are characterized by the occurrence of multiple contacts, almost independently of the moieties lining the site (i.e., the interacting groups can come from both backbone and side chains, and can be both polar and nonpolar). On average, a urea molecule is involved in five contacts, and a guanidinium ion or a thiocyanate ion is involved in six contacts; the three denaturants prove to be very promiscuous in their binding ability. These structural findings support a denaturant binding model to equal and independent sites. This does not mean that all aspects of this topic are clarified because our approach is limited by construction: it cannot provide the identity of the first places where denaturants bind nor information on the unfolding pathway of a protein [56].

On the other hand, our investigations highlight some interesting insights into the different denaturing power of these three compounds. A comparison of the data collected in the present study with those previously reported [28,29] indicates a reduced tendency of urea to interact with the π-system of aromatic residues with respect to both SCN^−^ and Gdm^+^ ions. As protein unfolding results in the exposure of the protein hydrophobic core that is also filled with aromatic side chains, protein unfolding leads to a larger increase of possible binding sites for thiocyanate and guanidinium ions, which, as a consequence, have a denaturing power stronger than that of urea.

In conclusion, the present study, which expands and complements recent surveys on the binding modes of denaturants to proteins [28,29,57], gives insights into the general mechanism of action of protein denaturants and clues into their different denaturing properties. 

## 5. Conclusions

A careful and extensive investigation of the Protein Data Bank provides structural hints on the denaturing action of the most frequently used denaturing agents. The promiscuity of interactions with all the components of protein chains is a common and fundamental trait for urea, guanidinium, and thiocyanate ions. In this context, we propose a general mechanistic model in which urea, due to its chemical features, proves to be the chemical agent endowed with the milder denaturing power.

## Figures and Tables

**Table 1:** The frequency of interactions established by ligands in our dataset of protein structures is reported as the percentage of total ligand binding sites (289 for urea, 127 for Gdm^+^, 712 for SCN^−^). Gdm^+^ and SCN^−^ related data were retrieved from refs [28,29].

Type of Interaction	Urea (%)	Gdm^+^ (%)	SCN^−^ (%)
H-bond with the protein	69.2	60.6	37.2
H-bond with main chain	45.0	48.0	14.3
H-bond with side chain	47.4	26.0	27.4
Salt bridge	-	44.1	29.4
van der Waals	88.6	96.9	95.6
Aliphatic interactions	84.8	91.3	92.7
Aromatic interactions	25.6	48.0	35.8
Interactions with cofactors/other ligands	3.8	7.9	7.2
Interaction with ion/metal	2.8	3.9	9.3
urea-Arg, Gdm^+^-Arg interactions	22.8	17.3	-
urea-urea, Gdm^+^-Gdm^+^, SCN^−^-SCN^−^	12.1	5.5	11.1
H-bond with water	65.4	67.7	50.0

## Data Availability

Protein structures were retrieved from the Protein Data Bank (https://www.rcsb.org/ (accessed on 31 May 2022)).

## Supplementary Materials

The following supporting information can be downloaded at: https://www.mdpi.com/article/10.3390/biology11121764/s1, Table S1: Ensemble of the 43 different protein types that bind urea molecules reported in the PDB; Table S2: Comparison among the distributions of the number of contacts per site established by the urea molecules and previously reported Gdm^+^ and SCN^−^ ligands. Figure S1: Urea structure; Figure S2: Distribution of urea concentration used in the analyzed 47 protein structures; Figure S3: Representative example of multiple types of interactions made by urea; Figure S4: Interactions with nonpolar groups; Figure S5: Distribution of water molecules per Gdm^+^ and SCN^−^ binding sites. References [28,29] are cited in the supplementary materials.
