# Supplementary material for: A Structure-Based Mechanism for the Denaturing Action of Urea, Guanidinium Ion and Thiocyanate Ion
Source: Biology (Basel). 2022 Dec 5;11(12):1764. doi: 10.3390/biology11121764 (PMC9775367; doi:10.3390/biology11121764)
Supplement: Supplementary file 1 [file biology-11-01764-s001.zip › biology-2035144-supplementary.pdf]

Supplementary Information

A structure-based mechanism for the denaturing action of urea, guanidinium ion and thiocyanate ion

Antonella Paladino [1*], Nicole Balasco [2], Luigi Vitagliano [1], Giuseppe Graziano [3*]

[1]Institute of Biostructures and Bioimaging, CNR, Via Pietro Castellino 111, 80131 Naples, Italy
[2]Institute of Molecular Biology and Pathology, CNR c/o Dep. Chemistry, University of Rome, Sapienza, P.le A. Moro 5, 00185 Rome, Italy.
[3]Department of Science and Technology, University of Sannio, via Francesco de Sanctis snc, 82100 Benevento, Italy

Tables

Table S1. Ensemble of the 47 different protein types that bind urea molecules reported in the PDB. The PDB entry, the resolution of the structure, the protein molecule, the number of urea molecules and chains present in the asymmetric unit (A.U.), the biological assembly (B.A.) and the number of independent urea molecules are reported. PDB entries whose asymmetric unit contains more or less copies of the biological assembly are indicated in red and blue, respectively.

PDB entry	Resolution (Å)	Protein name	Urea in A.U.	Chains in A.U.	B.A.	Independent urea	Urea concentration (M)*
1BV3	1.85	Carbonic Anhydrase	1	1	monomeric	1	>0.1
1DDR	2.45	Dihydrofolate Reductase	5	2	dimeric	5	2.45
1EP5	2.30	Capsid Protein	7	3	trimeric	7	NA
1HQG	2.00	Arginase 1	3	3	trimeric	1	NA
1XDQ	2.55	Bacterial Oxidoreductase	5	5	monomeric	1	1
1XR9	1.79	HLA-B	1	2	dimeric	1	8
2F56	1.96	Ribonuclease	11	3	monomeric/ dimeric	11	6
2I1J	2.10	Moesin	2	1	monomeric	2	1
2J8X	2.30	Uracil-DNA Glycosylase	2	4	dimeric	1	NA
2OM0	2.05	Insulin	12	36	dodecameric	10	3
2Q8V	2.50	NblA	2	2	dimeric	2	2
2R2X	2.40	Ricin A	1	1	monomeric	1	1
2XLA	1.93	SLL1785	4	4	dimeric	1	NA
3EP5	1.99	AMD1	1	2	dimeric	1	NA
3L4P	1.45	BamD	1	1	monomeric	1	NA
3MFF	2.00	TCR	3	4	dimeric	3	NA
3MSF	2.09	Thermolysin	1	1	monomeric	1	<0.1
3NLS	1.70	Protease	1	2	dimeric	1	NA

3P06	2.10	VP4 protein	1	1	monomeric	1	0.2
3RPW	1.65	ABC Transporter	1	1	monomeric	1	6
3TPP	1.60	beta-Secretase 1 – BACE1	11	1	monomeric	11	0.5
3UB6	1.38	Chemoreceptor TipB	2	2	dimeric	1	NA
3UMV	1.71	Deoxyribopyrimidine Photolyase	9	2	monomeric	5	NA
3VA7	2.60	Carboxylase	1	1	monomeric	1	NA
3ZYR	1.65	Lectin	2	2	dimeric	2	8
4CE8	0.90	Fucose binding Lectin II	1	4	tetrameric	1	NA
4EVA	2.00	Catenin beta-1	72	2	monomeric	64	5.6
4FR8	2.20	ALDH2	6	8	tetrameric	3	0.06
4GY3	2.50	C-phycocianinB	19	2	hexameric	19	2
4OMX	2.30	Beta-LG	3	1	dimeric	3	NA
4TPU	2.35	Rubredoxin	2	1	trimeric	2	0.1
4U4M	3.09	YagE	10	4	tetrameric	7	0.5
5APU	1.35	GCN4	1	3	trimeric	1	1
5AUN	1.63	HypA	3	2	tetrameric	3	NA
5I4Y	1.61	Lysozyme C	21	1	monomeric	21	9
5II0	2.10	Calcitonin Receptor	1	3	monomeric	1	2
5NI9	1.33	HLA-DRB1	2	3	trimeric	2	8
5NXF	1.90	Long-tail fiber proximal subunit	1	3	trimeric	1	NA
5ULP	1.55	MethylTransferase	8	2	monomeric	8	8
6EWN	2.29	HspA	44	2	monomeric	44	2
6FEJ	2.75	All4940 protein	3	2	monomeric	3	1
6NFP	1.70	Arginase	16	6	hexameric	16	NA
6QDY	1.42	Urease	1	3	nonameric	1	0.2
7S6F	1.80	Putative urea ABC transporter UrtA1	1	1	monomeric	1	NA
7A36	1.50	Proto-oncogene tyrosine-protein Kinase SRC	13	2	monomeric	13	7
7JOW	1.91	Kallikrein 4	1	2	dimeric	1	8
7LOL	1.80	Agmatinase	1	1	hexameric	1	NA

*The urea molar concentration was extracted from the PDB file (remark 280 crystallization conditions) or from the related paper, neither numerical derivation nor experimental approximation was made. NA stands for not available data.

Table S2. Comparison among the distributions of the number of contacts *per* site established by the urea molecules and previously reported Gdm^+ and SCN^- ligands [1,2].

Number of contacts *per* site	Number of urea ligands	Number of Gdm^+ ligands	Number of SCN^- ligands
0	5	0	6
1	26	0	22
2	32	10	47
3	35	9	67
4	43	13	90
5	29	10	123
6	29	26	125
7	36	19	99
8	27	18	79
9	16	7	38
10	8	9	11
11	1	6	4
12	0	0	1
13	2	0	0

Figures

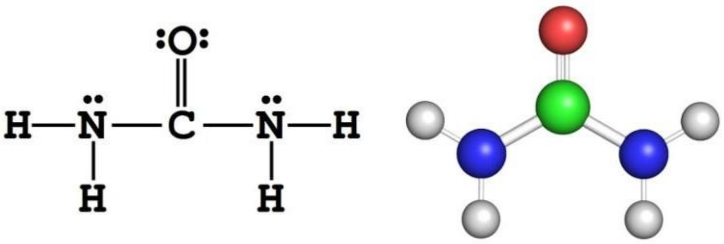

Figure S1. Urea molecule: Lewis Structure and ball&stick representation.

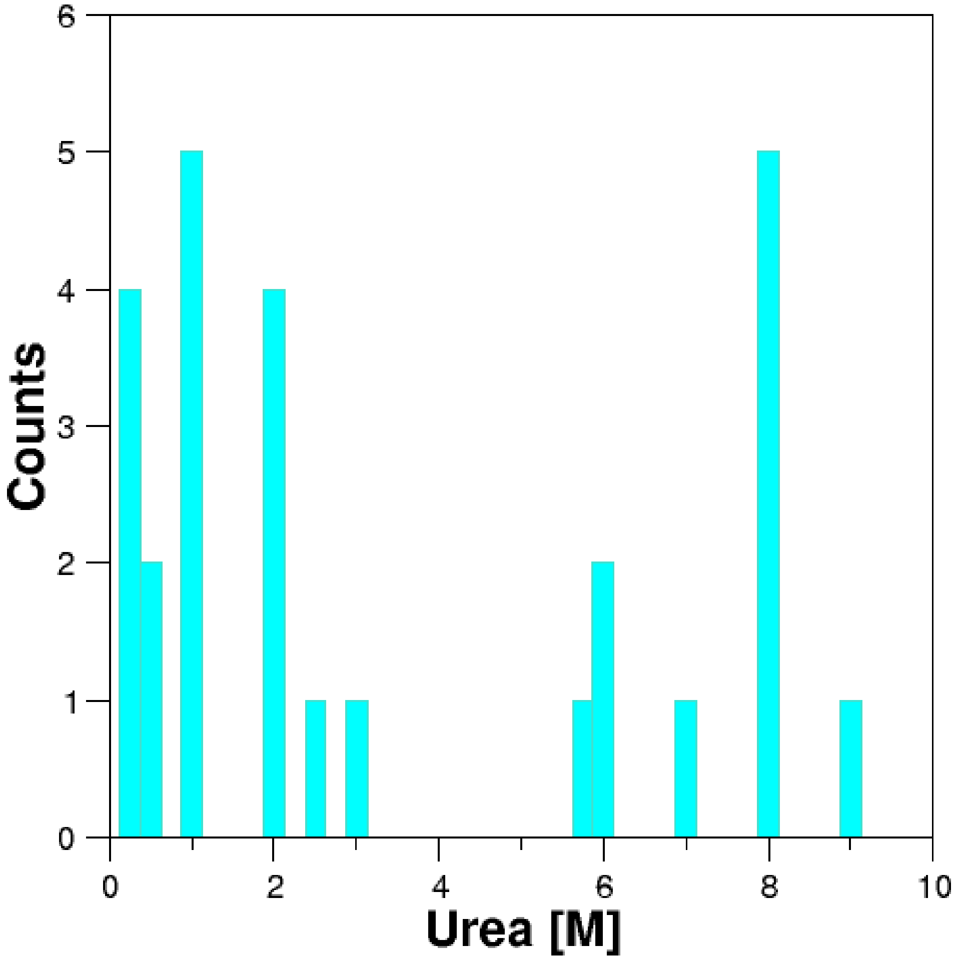

Figure S2. Distribution of urea molar concentration. Data (available for 27 out of the 47 protein structures analyzed) were extracted from the PDB file (remark 280 crystallization conditions) or from the related paper (see Table S1).

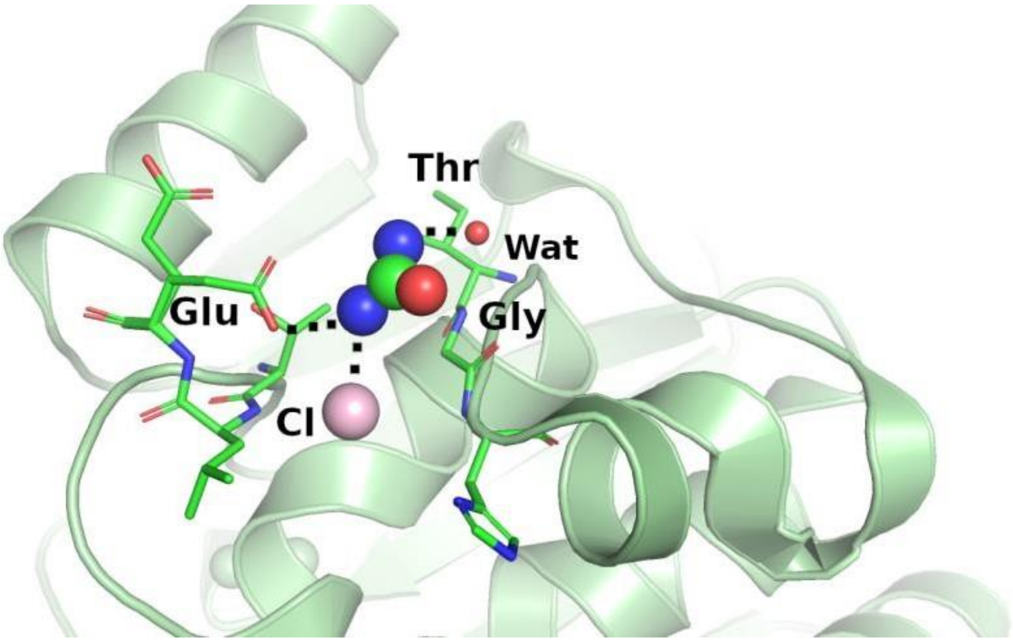

Figure S3. Interaction network where urea makes multiple type of contacts, namely H-bonds, aliphatic and ion interactions (PDB ID: 6NFP).

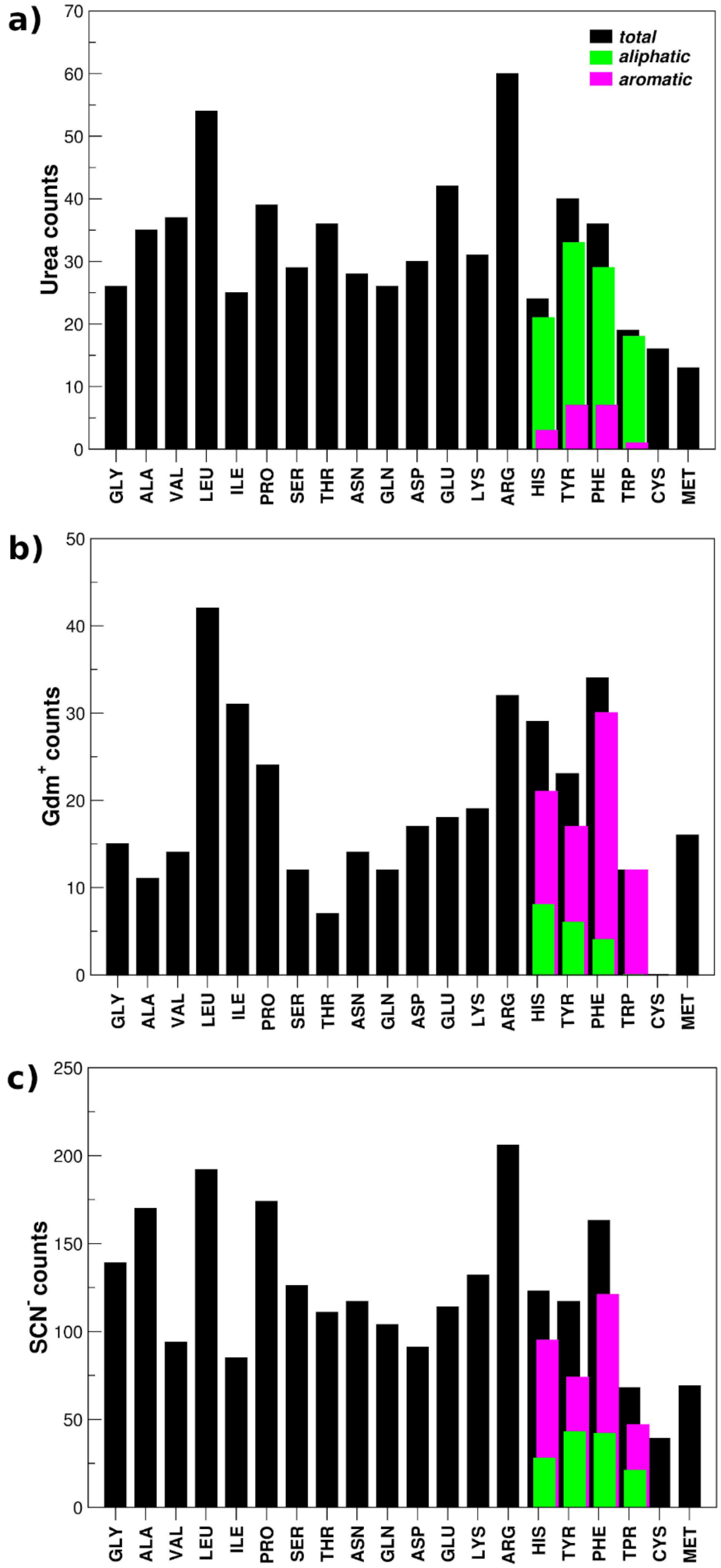

Figure S4. Interactions with nonpolar groups. Representativeness of residue type involved in hydrophobic interactions per Urea (a), Gdm^+ (b) and SCN^- (c) sites. Data relative to SCN^- are adapted from reference [2] and distribution referred to Gdm^+ is generated using database of reference [1].

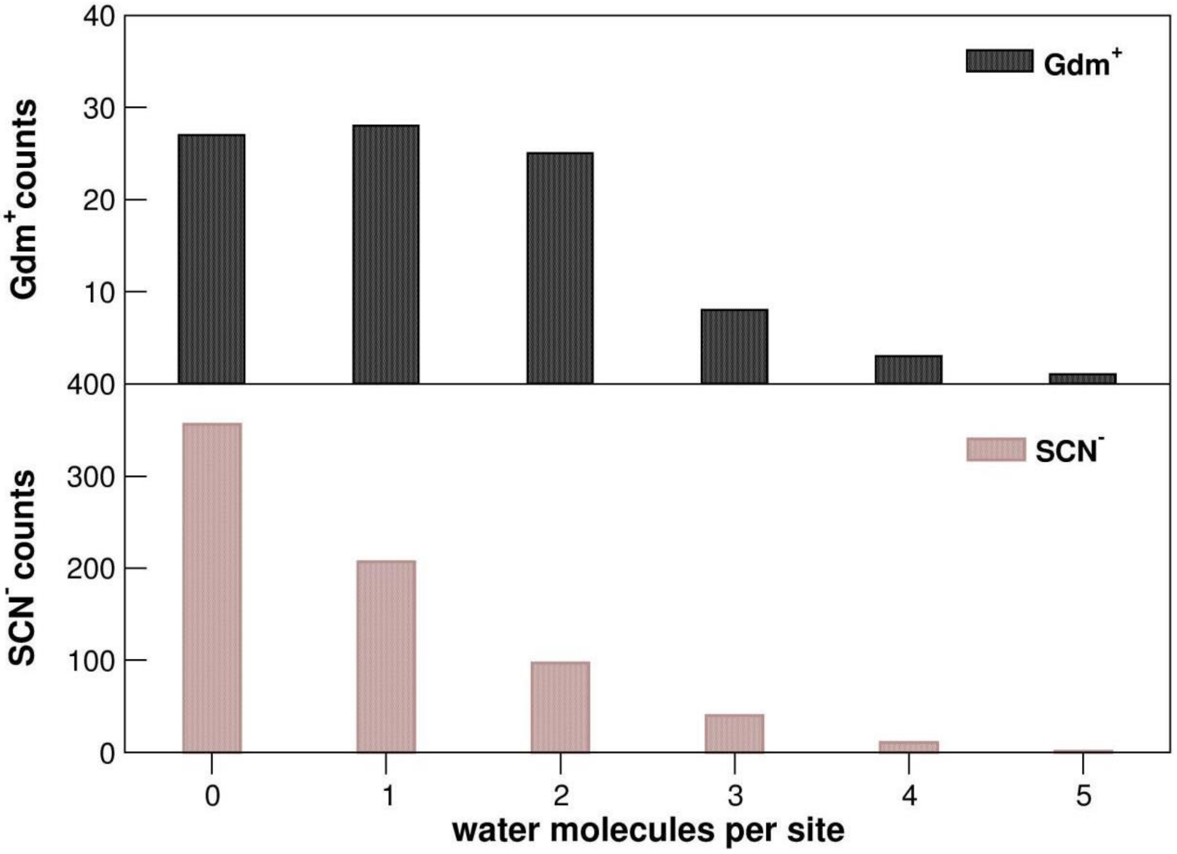

Figure S5. Distribution of water molecules per Gdm+ and SCN- binding sites. Data relative to SCN^- and Gdm^+ are adapted from reference [1,2].

References

[1] S. Cozzolino, N. Balasco, M. Vigorita, A. Ruggiero, G. Smaldone, P. Del Vecchio, L. Vitagliano, G. Graziano, Guanidinium binding to proteins: The intriguing effects on the D1 and D2 domains of Thermotoga maritima Arginine Binding Protein and a comprehensive analysis of the Protein Data Bank, International Journal of Biological Macromolecules. 163 (2020) 375–385. https://doi.org/10.1016/j.ijbiomac.2020.06.290.

[2] A. Paladino, N. Balasco, G. Graziano, L. Vitagliano, A Protein Data Bank survey of multimodal binding of thiocyanate to proteins: Evidence for thiocyanate promiscuity, International Journal of Biological Macromolecules. 208 (2022) 29–36. https://doi.org/10.1016/j.ijbiomac.2022.03.012.
